# Neuroplasticity in Spinal Circuits Mediated by Sexual Experience and Cerebellar Lobules

**DOI:** 10.3390/neurosci7030067

**Published:** 2026-06-11

**Authors:** Jaime R. Gutiérrez, Cristofer Zarate-Calderon, Fiorella Fadanelli-Sánchez, Abdiel A. Demuner-Mendoza, René Zempoalteca-Ramírez, Luis Beltrán Parrazal, Donaji Chi-Castañeda, Luis I. García

**Affiliations:** 1 Instituto de Investigaciones Cerebrales, Universidad Veracruzana, Xalapa 91190, Mexico; 2 Facultad de Medicina, Campus Ciudad Mendoza, Universidad Veracruzana, Ciudad Mendoza 94740, Mexico; 3 Centro Tlaxcala de Biología de la Conducta, Universidad Autónoma de Tlaxcala, Tlaxcala 90070, Mexico

**Keywords:** H-reflex, cerebellum, motor learning, sexual experience, spinal cord

## Abstract

Objective: We aimed to determine whether sexual experience modulates the soleus H-reflex in male rats and to assess the specific contribution of vermis lobules 6a and 7 to cerebellar-dependent spinal plasticity. Methods: Thirty-six male Wistar rats were divided into sexually inexperienced (SI) and sexually experienced (SE) groups and assigned to one of three cerebellar conditions: intact control, lobule 6a lesion, or lobule 7 lesion. SE rats underwent repeated mating sessions until they achieved efficient copulatory performance. Subsequently, targeted electrolytic lesions were made, and electromyographic recordings of the soleus H-reflex were obtained under urethane anesthesia to quantify H-wave amplitude and temporal parameters. Results: The global linear mixed model yielded no significant main effects of sexual experience, cerebellar condition, or their interaction on H-wave amplitude. Planned contrasts revealed a near-significant trend toward higher H-wave amplitude in sexually experienced intact animals compared with inexperienced controls (*p* = 0.061, Cohen’s d = 0.592, 95% CI [−1.44, 0.04] V), and significant amplitude reductions following lobule 6a (*p* = 0.029, d = 1.450, 95% CI [0.13, 2.33] V) and lobule 7 (*p* = 0.002, d = 2.256, 95% CI [0.74, 3.08] V) lesions specifically in sexually experienced animals. Neither sexual experience nor lesions significantly affected H-wave latency or duration, suggesting that modulation primarily targets synaptic excitability rather than axonal conduction. M-wave latency showed a significant effect of sexual experience (*p* = 0.026, d = 1.405, 95% CI [0.03, 0.45] ms). Conclusions: Sexual experience appears to be associated with cerebellar-dependent modulation of soleus H-reflex excitability; lobules 6a and 7 of the cerebellar vermis contribute to this effect specifically in experienced animals. Shorter M-wave latency in experienced animals suggests parallel peripheral motor reorganization. Adequately powered confirmatory studies are needed to characterize the mechanisms underlying this association.

## 1. Introduction

The cerebellum is a structure extensively related to sensorimotor integration, crucial for the acquisition and precise execution of movements and behaviors [1,2,3]. Alongside its contributions to coordination and posture, its descending projections modulate the activity of spinal neurons involved in reflexes, locomotion, and complex movements through reticulospinal and vestibulospinal pathways, complementing the voluntary motor commands of the pyramidal system [4].

This structure plays a crucial role not only in motor execution but also in motor learning. Cerebellar–cortical circuits are fundamental to this process, as alterations in synaptic efficacy between parallel fibers and Purkinje cells, modulated by error signals transmitted by climbing fibers, allow for the correction and refinement of movements [5,6,7,8].

Among the basic motor responses coordinated at the spinal level is the stretch or myotatic reflex, an immediate muscular response to sudden stretch, mediated mainly by a monosynaptic pathway involving the group Ia afferent fiber originating from the primary endings of the muscle spindle and its direct synaptic connection with the alpha motoneuron [9,10,11]. A tool for studying these pathways in humans and animal models is the H-reflex, which allows evaluation of monosynaptic connections between sensory fibers and spinal motoneurons by electrically stimulating a peripheral nerve and recording the reflex response in the homonymous muscle [12].

In this context, it has been demonstrated that the cerebellum is essential for acquiring and maintaining conditioned depression of the H-reflex, a motor learning paradigm. Animal studies have shown that lesions in the interpositus and dentate nuclei of the cerebellum result in the loss of this previously acquired learning, underscoring cerebellar involvement in the plasticity of spinal circuits [4,13,14].

In animal models, sexually experienced male rats show increased expression of Fos protein, a marker of neuronal activity, in granule cells of lobules 6–9 of the cerebellar vermis. A biphasic activation–deactivation pattern has been observed in granule neurons of the medial vermis during sexual behavior, suggesting a process that correlates with motor performance and the sensory stimulation inherent to copulation [14]. Additionally, electrophysiological studies have implicated lobules 6a and 7 of the vermis, as well as the fastigial nucleus and the inferior olive, in modulating sexual behavior and its associated motor learning [15,16]. Crucially, experience-dependent cerebellar plasticity has been documented broadly in animal models, with enriched behavioral stimulation producing measurable changes in cerebellar structure and function [17], and sexual experience, in particular, inducing micro- and macrostructural neuroplastic changes across multiple brain regions [18].

Despite evidence linking the cerebellum to motor learning and H-reflex modulation, and the establishment of sexual behavior as a motor learning model involving specific cerebellar regions, it remains unclear how acquiring sexual experience in male rats modifies the amplitude or latency of the H-reflex. This knowledge gap justifies the use of sexual learning as a natural paradigm for procedural memory that induces a reconfiguration of cerebellar circuits.

Therefore, the objective of the present work is to characterize the H-reflex of the soleus muscle in sexually inexperienced and experienced male rats, and to determine the effect that electrolytic lesion of lobules 6a and 7 of the cerebellar vermis has on this reflex in both groups. This research seeks to elucidate the neuroplastic changes in cerebello-spinal pathways that underlie motor learning associated with sexual experience.

## 2. Materials and Methods

### 2.1. Subjects and Housing Conditions

Male (*n* = 36) and female (*n* = 6) Wistar rats were used, with a body weight of 250–350 g and a postnatal age of 60–80 days, obtained from a breeding colony at the Institute of Brain Research, Universidad Veracruzana. Animals were housed individually in transparent acrylic boxes (44 × 34 × 20 cm) with sterile sawdust bedding (Harlan, Mexico), under a 12 h inverted light–dark cycle (lights off from 7:30 to 19:30 h CST) and with ad libitum access to standard food and water. All procedures adhered to the Official Mexican Standard NOM-062-Z00-1999 guidelines [19]. Ethical approval was granted by the Internal Committee for the Care and Use of Laboratory Animals of the Brain Research Center (approval number CICUAL-CICE-2017-018).

The inclusion criteria were Wistar rats that met the criteria for sex, weight, and age at the beginning of the experiment and had not been previously exposed to experimental or pharmacological procedures. Additionally, animals had to complete the scheduled recording and sexual experience sessions without showing signs of illness, discomfort, or surgical complications that could prevent continuation of the study.

To ensure adequate allocation of male rats, a random number generator was used to assign animals to the established groups: sexually inexperienced (SI) (*n* = 18) and sexually experienced (SE) (*n* = 18). Animals in group SI had no sexual exposure. Animals in the SE group were trained until they showed efficient copulatory behavior.

Subsequently, both animals from groups SI and SE were randomly subdivided into three subgroups (*n* = 6 each):

1. Intact Control: Without cerebellar surgical intervention, where only baseline H-reflex measurements were recorded.
2. L6a Lesion: Subjected to an electrolytic lesion of lobule 6a of the cerebellar vermis.
3. L7 Lesion: Subjected to an electrolytic lesion of lobule 7 of the cerebellar vermis.

### 2.2. Training for Sexual Experience Acquisition

Males from the SE group were subjected to a maximum of four training sessions to induce sexual experience. Each session was conducted in circular arenas (80 cm diameter) with sawdust bedding, under red light. After a habituation period (10 min to the room and 5 min to the arena), a sexually receptive female was introduced. Behavioral parameters, such as the number of mounts, intromissions, and latency to ejaculation, were recorded. Sessions concluded with male ejaculation or after a maximum period of 30 min without definitive copulatory activity.

The criterion for classification as sexually experienced was the achievement of ejaculation in at least three independent training sessions. Animals that failed to show intromission within the first 30 min of any session were classified as non-copulators (NC), while those that showed intromission but did not achieve ejaculation within 30 min after the first intromission across sessions were classified as poor copulators (PC); both NC and PC animals were excluded from the SE group and not subjected to further experimental procedures. In each session, the number of mounts, intromissions, and ejaculations was recorded, along with mount latency, intromission latency, and ejaculation latency. Therefore, the sexually experienced group, as constituted, comprises males with efficient copulatory performance; animals with lower copulatory capacity were not further studied.

The females used were bilaterally ovariectomized under general anesthesia (*n* = 6). Sexual receptivity was pharmacologically induced through subcutaneous administration of estradiol benzoate (10 µg in 0.1 mL of vegetable oil) 48 h before, and progesterone (2 mg in 0.1 mL of vegetable oil) 4 h before each test session.

### 2.3. Cerebellar Lesion Procedure

Animals assigned to lesion groups were anesthetized with urethane (2.0 g/kg, i.p., 20% solution). Urethane was selected as the anesthetic agent because it acts on multiple neurotransmitter receptor systems simultaneously: it potentiates nicotinic, GABAergic, and glycinergic receptors while modestly inhibiting NMDA and AMPA-receptor-mediated currents, without the preferential GABAergic enhancement characteristic of barbiturates. This balanced pharmacological profile preserves spinal monosynaptic reflexes with minimal disruption and maintains neural connectivity patterns close to the unanesthetized state, making it the standard agent for acute H-reflex recordings in rats [20]. Its long duration of action ensures stable anesthetic depth throughout the combined lesion and recording session without supplemental dosing. Once the anesthetic plane was confirmed by the absence of palpebral reflex and pedal withdrawal response to firm toe pinch or by checking the absence of the flexor reflex in the lower limbs, the animal was placed in a stereotaxic apparatus (Stoelting Co. 51670, Wood Dale, IL, USA).

Using coordinates from the Paxinos and Watson atlas [21], an insulated stainless steel monopolar electrode (FHC, Inc., Bowdoin, ME, USA) was lowered to the cortex of lobule 6a (AP: −12.0 mm from bregma; ML: 0.0 mm; DV: −1.9 mm) and lobule 7 (AP: −12.6 mm from bregma; ML: 0.0 mm; DV: −3.8 mm) of the cerebellar vermis, using a hydraulic micropositioner (Kopf Instruments, Tujunga, CA, USA).

The lesion was induced by applying anodal direct current (3.5 mA for 20 s) through a stimulator (S48, Grass Technologies, Warwick, RI, USA) and a direct current unit (CCU1A, Grass Technologies, Warwick, RI, USA). These parameters were previously standardized in pilot tests to ensure selective ablation of areas of interest with minimal effects on surrounding tissue and animal survival. After electrode removal, the incision was sutured.

Subsequently, lesion placement was histologically verified in all animals at the end of the experimental session. Animals were transcardially perfused with heparinized 0.9% saline, followed by 4% paraformaldehyde in 0.1 M phosphate buffer (pH 7.4). Cerebella were extracted, post-fixed for 24 h at 4 °C, cryoprotected in 30% sucrose, and sectioned transversally at 20 µm on a cryostat (Leica Microsystems CM1850, Nussloch, Germany). Sections were stained with cresyl violet (Sigma-Aldrich, St. Louis, MO, USA; C5042) and examined under optical microscopy (Olympus Corporation AX70, Tokyo, Japan) to confirm lesion placement within the target lobule and assess the extent of tissue disruption. All lesioned animals included in the final analysis showed tissue damage confined to the target lobule, with no evidence of spreading to adjacent vermis lobules or deep cerebellar nuclei.

### 2.4. Electromyographic Recording of H-Reflex

Immediately after lesion surgery, H-reflex recording was performed while maintaining the same urethane anesthesia. Adequate anesthetic depth was monitored throughout the recording session by periodic reassessment of palpebral and pedal withdrawal reflexes. With the animal in dorsal decubitus, the ipsilateral tibial nerve and soleus muscle were surgically exposed. The tibial nerve was carefully isolated in the popliteal fossa.

Bipolar hook electrodes, fabricated from chloride silver wire and insulated except at the contact tip, were used for tibial nerve stimulation, with the electrode pair covered with mineral oil to prevent desiccation and ensure electrical isolation. For electromyographic (EMG) recording, fine wire electrodes (exposed tip ~1.5 mm, separated by >5 mm) were inserted into the soleus muscle belly. A ground electrode was placed in the adjacent subcutaneous tissue.

The H-reflex was evoked through tibial nerve stimulation. Recruitment curves for H and M waves were obtained by progressively increasing stimulus intensity (single pulses) until the maximum M wave response was observed. Stimuli were delivered at an inter-stimulus interval of ≥10 s to prevent post-activation depression of the H-reflex, ensuring that each trial reflected steady-state motoneuron pool excitability rather than a recovery-dependent transient.

Sixteen EMG responses were averaged for each stimulus intensity. The signal was amplified and filtered using a band-pass configuration (high-pass: 100 Hz; low-pass: 1000 Hz; Software Link15), and visualized/recorded using a digital system (Polyview viewer, GRASS; PVA-16 adapter; West Warwick, RI, USA) and an oscilloscope (TDS 3012C Tektronix, Inc., Beaverton, OR, USA), both synchronized with the stimulator.

Prior to averaging, individual traces were visually inspected; those presenting stimulus artifact contaminating the H-wave time window or baseline noise exceeding the amplitude of the smallest detectable H-wave were excluded. The remaining traces were retained irrespective of waveform morphology to avoid selection bias in the averaged response.

### 2.5. Analysis Procedure

#### 2.5.1. Design and Variables

A 2 × 3 factorial design with repeated measures was employed to evaluate the effects of sexual experience (inexperienced vs. experienced) and cerebellar condition (Intact, L6a Lesion, L7 Lesion) on H-reflex parameters. The dependent variables were onset latency (ms), duration (ms), and peak-to-peak amplitude (V) of M and H waves. The electrical stimulus intensity (µA) was included as a continuous covariate and standardized prior to model fitting.

#### 2.5.2. Statistical Models

Given the hierarchical structure of the data (multiple records per rat, rats nested within groups), Linear Mixed Models were utilized. For each dependent variable, the following model was fitted:

$$Y_{ijk}=\;\beta_{0}+\;\beta_{1}\left(SE_{i}\right)+\beta_{2}\left({CC}_{j}\right)+\beta_{3}\left(SE_{i}\times {CC}_{j}\right)+\beta_{4}\left(I_{ijk}\right)+u_{k}+\epsilon_{ijk}$$

where ${SE}_{i}$ refers to sexual experience, ${CC}_{j}$ is the cerebellar condition, $I_{ijk}$ is the intensity, $u_{k}$ represents the random effect of the rat $k\sim N(0,\sigma_{u}^{2})$ and $\epsilon_{ijk}$ represents the residual error $\sim N(0,\sigma_{\epsilon }^{2})$.

Models were fitted using Restricted Maximum Likelihood (REML). The significance of fixed effects was evaluated with Type III ANOVA using Satterthwaite’s degrees of freedom approximation. Model selection between models with and without interaction was performed using likelihood ratio tests (α = 0.05).

*Post hoc* comparisons were conducted using estimated marginal means with Tukey’s HSD correction. Planned contrasts were performed to test specific a priori hypotheses: (H1) effect of sexual experience in intact animals, and (H2) effect of cerebellar lesions on learned facilitation. Contrast H1 was estimated from a submodel restricted to intact animals, and H2 from a submodel restricted to sexually experienced animals, to obtain variance estimates specific to each hypothesis. Effect sizes were reported as Cohen’s d (|estimate| / σ) and 95% confidence intervals were derived using the Kenward–Roger method.

The global LMM, which includes all six experimental groups simultaneously, has limited statistical power to detect subgroup-specific effects. The planned contrasts address this by fitting restricted submodels with variance estimates tailored to each hypothesis, thereby constituting the primary confirmatory tests for H1 and H2. Model diagnostics, including residual normality, homoscedasticity, and normality of random effects, were assessed using the car package and confirmed adequate model fit. Random slopes for intensity were evaluated by likelihood ratio test and did not improve model fit (*p* > 0.05), supporting the random-intercept-only structure. The Satterthwaite degrees of freedom for between-group effects (df = 28–32) correspond to the animal-level sample size, confirming that repeated within-animal measurements do not inflate type I error for between-group comparisons.

Model fit quality was evaluated using marginal R^2^ (variance explained by fixed effects) and conditional R^2^ (fixed + random effects). The Hmax/Mmax ratio was calculated per animal as the maximum H-wave amplitude divided by the maximum M-wave amplitude across all recorded intensities, and group differences were evaluated using one-way ANOVA.

All statistical analyses were performed in R (Version 4.4.3, R Foundation for Statistical Computing, Vienna, Austria) within the RStudio environment for Windows (Version 2024.12.1 + 563), using the lme4 (mixed models), lmerTest (significance tests), emmeans (*post hoc* comparisons), car (homoscedasticity tests), and performance (model evaluation) packages. α = 0.05 was established for all hypothesis tests.

## 3. Results

### 3.1. Descriptive Statistics

A total of 385 average electrophysiological records (equivalent to 6160 individual records) were considered, distributed across six experimental groups: three without sexual experience under different cerebellar conditions, including intact controls, and three with sexual experience under the same cerebellar conditions. The analysis focused on determining latency, duration, and amplitude.

**Figure 1:**
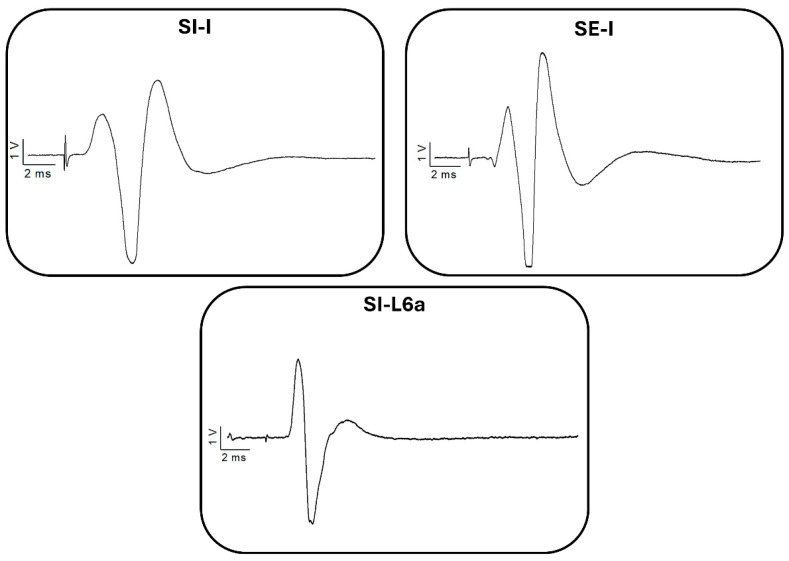
Representative EMG recordings of M- and H-waves from the soleus muscle under different experimental conditions. Traces correspond to three experimental groups: SI-I: sexually inexperienced intact rats; SE-I: sexually experienced intact rats; and SI-L6a: sexually inexperienced rats with an electrolytic lesion of cerebellar vermis lobule 6a. Calibration: vertical = 1 V; horizontal = 2 ms.

Characteristic EMG recordings of M- and H-waves were obtained from the soleus muscle under the different experimental conditions. Figure 1 illustrates representative traces from three key groups: sexually inexperienced intact rats (SI-I), sexually experienced intact rats (SE-I), and sexually inexperienced rats with an electrolytic lesion of cerebellar vermis lobule 6a (SI-L6a).

### 3.2. Latency

**Figure 2:**
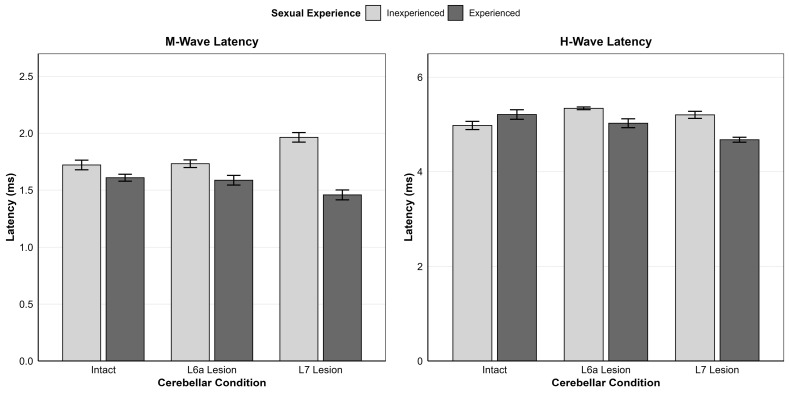
Onset latency of M-wave and H-wave responses across cerebellar conditions and sexual experience groups. (**Left**) panel: M-wave latency (ms). (**Right**) panel: H-wave latency (ms). X-axis: cerebellar condition. M-wave latency showed a significant main effect of sexual experience (F(1,28.44) = 5.49, *p* = 0.026), with inexperienced animals displaying longer latencies than experienced animals across all cerebellar conditions (estimated difference = +0.24 ms); H-wave latency remained unaffected by experimental manipulations. Error bars represent SEM.

Analysis of M-wave latency showed significant effects for sexual experience (F(1,28.44) = 5.49, *p* = 0.026) and stimulus intensity (F(1,355.70) = 12.00, *p* < 0.001), but not for cerebellar condition (F(2,28.27) = 0.58, *p* = 0.568) or their interaction (F(2,28.22) = 1.78, *p* = 0.187) (Figure 2). The model explained 16.7% of the variance (marginal R^2^) and 77.8%, including random effects (conditional R^2^). Planned contrast indicated that sexually inexperienced animals showed longer M-wave latency than experienced animals (estimate = +0.24 ms, 95% CI [0.03, 0.45], t(28.3) = 2.343, *p* = 0.026, Cohen’s d = 1.405), with marginal means of 1.80 ms and 1.56 ms, respectively.

On the other hand, H-wave latency showed no significant effects of sexual experience (F(1,27.63) = 0.22, *p* = 0.645), cerebellar condition (F(2,27.51) = 0.21, *p* = 0.812), or their interaction (F(2,27.48) = 0.60, *p* = 0.557). Stimulus intensity showed a significant effect (F(1,354.08) = 3.88, *p* = 0.050). The model explained 7.7% of the variance (marginal R^2^) and 80.9% of the variance, including random effects.

### 3.3. Duration

**Figure 3:**
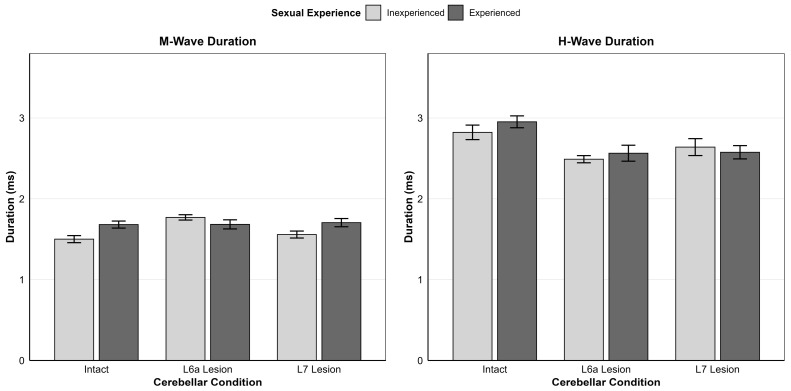
Duration of M-wave and H-wave responses across cerebellar conditions and sexual experience groups. (**Left**) panel: M-wave duration (ms). (**Right**) panel: H-wave duration (ms). X-axis: cerebellar condition. Neither M-wave nor H-wave duration showed significant effects of sexual experience, cerebellar condition, stimulus intensity, or their interaction. Error bars represent SEM.

Analysis of M-wave duration showed no significant effects for sexual experience (F(1,28.16) = 0.82, *p* = 0.372), cerebellar condition (F(2,28.00) = 0.82, *p* = 0.449), stimulus intensity (F(1,355.40) < 0.001, *p* = 0.995), or their interaction (F(2,27.95) = 0.61, *p* = 0.551) (Figure 3). The model explained 8.1% of the variance (marginal R^2^) and 76.0% including random effects (conditional R^2^).

For H-wave duration, no significant effects were found for sexual experience (F(1,27.82) = 1.02, *p* = 0.321), cerebellar condition (F(2,27.64) = 1.04, *p* = 0.366), stimulus intensity (F(1,355.46) = 1.67, *p* = 0.198), or their interaction (F(2,27.59) = 0.01, *p* = 0.986). The model explained 5.8% of the variance (marginal R^2^) and 73.6% including random effects.

### 3.4. Amplitude

**Figure 4:**
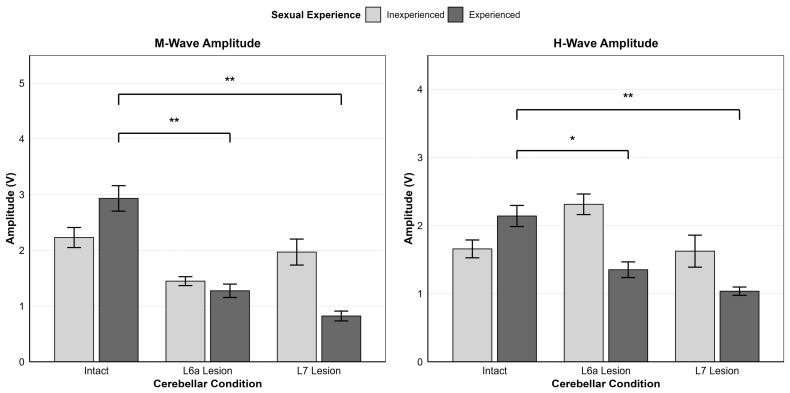
Peak-to-peak amplitude of M-wave and H-wave responses across cerebellar conditions and sexual experience groups. (**Left**) panel: M-wave amplitude (V). (**Right**) panel: H-wave amplitude (V). X-axis: cerebellar condition. M-wave amplitude showed a significant main effect of cerebellar condition (F(2,30.16) = 8.63, *p* = 0.001); brackets indicate significant *post hoc* comparisons within the sexually experienced group: SE-Intact vs. SE-L6a Lesion (*p* = 0.007) and SE-Intact vs. SE-L7 Lesion (*p* = 0.003). H-wave amplitude showed no significant omnibus effects; planned a priori contrasts revealed significant reductions in sexually experienced animals following lobule 6a lesion (SE-Intact vs. SE-L6a: *p* = 0.029) and lobule 7 lesion (SE-Intact vs. SE-L7: *p* = 0.002). Error bars represent SEM. * *p* < 0.05; ** *p* < 0.01.

Analysis of M-wave amplitude revealed a significant effect of cerebellar condition (F(2,30.16) = 8.63, *p* = 0.001) and stimulus intensity (F(1,363.21) = 15.49, *p* < 0.001). No significant effects of sexual experience were observed (F(1,30.69) = 0.77, *p* = 0.388), although the interaction showed a trend (F(2,30.03) = 3.15, *p* = 0.057) (Figure 4). The model explained 23.1% of the variance (marginal R^2^) and 60.5% including random effects. *Post hoc* comparisons for the main effect of cerebellar condition revealed that intact animals showed significantly higher M-wave amplitude than both L6a-lesioned (estimate = 1.786 V, 95% CI [0.691, 2.881], *p* = 0.001, Cohen’s d = 1.795) and L7-lesioned animals (estimate = 1.222 V, 95% CI [0.129, 2.314], *p* = 0.026, Cohen’s d = 1.228), while L6a and L7 lesion groups did not differ from each other (*p* = 0.454). Stratified analysis indicated that these reductions were significant within the experienced group (Intact vs. L6a: *p* = 0.007, d = 1.957; Intact vs. L7: *p* = 0.003, d = 2.285) but did not reach significance within the inexperienced group (Intact vs. L6a: *p* = 0.051; Intact vs. L7: *p* = 0.960), consistent with the interaction trend.

For H-wave amplitude, no significant effects were found for sexual experience (F(1,31.65) = 0.09, *p* = 0.766), cerebellar condition (F(2,31.16) = 1.44, *p* = 0.252), or their interaction (F(2,31.03) = 1.00, *p* = 0.378). Stimulus intensity showed a significant effect (F(1,362.80) = 21.69, *p* < 0.001). The model explained 14.8% of the variance (marginal R^2^) and 58.6% including random effects.

### 3.5. Effect of Experience in Intact Animals

In the analysis restricted to intact animals (*n* = 12), the comparison between inexperienced and experienced groups showed a statistical trend that did not reach conventional significance (t(9.11) = −2.133, *p* = 0.061; 95% CI [−1.44, 0.04]; Cohen’s d = 0.592), with an estimated difference of −0.70 V.

### 3.6. Effect of Lesions in Experienced Animals

In the analysis of experienced animals (*n* = 18), significant differences were found between cerebellar conditions. *Post hoc* comparisons showed significant differences between Intact and L6a Lesion (t(14.0) = 2.913, *p* = 0.029) and between Intact and L7 Lesion (t(15.4) = 4.218, *p* = 0.002). The difference between L6a Lesion and L7 Lesion was not significant. The analysis results are shown in Table 1.

### 3.7. Hmax/Mmax Ratio Analysis

The Hmax/Mmax ratio was calculated per animal as the maximum H-wave amplitude divided by the maximum M-wave amplitude across all recorded stimulus intensities; complete Hmax and Mmax data were available for 33 of 36 animals. One-way ANOVA revealed no significant differences among the six experimental groups (F(5,27) = 0.688, *p* = 0.637). Group means (±SD) were as follows: SI-, 1.344 ± 0.834; SE-I, 0.970 ± 0.363; SI-L6a, 1.290 ± 0.650; SE-L6a, 1.573 ± 1.215; SI-L7, 0.818 ± 0.275; and SE-L7, 1.200 ± 0.796.

## 4. Discussion

The present work focused on determining whether sexual experience modulates the soleus H-reflex in male rats and whether lobules 6a and 7 of the cerebellar vermis contribute to that modulation. The patterns obtained are more nuanced than simple confirmation. When all six groups were analyzed together, sexual experience had no significant main effect on H-wave amplitude, nor did cerebellar condition. The signal emerged instead from the comparisons the study was designed to make: in intact animals, sexually experienced rats showed a trend toward larger H-wave amplitudes than inexperienced controls (*p* = 0.061, d = 0.592, 95% CI [−1.44, 0.04] V), and within sexually experienced animals, lesioning either lobule sharply reduced that amplitude (L6a: *p* = 0.029, d = 1.450, 95% CI [0.13, 2.33] V; L7: *p* = 0.002, d = 2.256, 95% CI [0.74, 3.08] V). We therefore interpret these results as preliminary but consistent evidence that the cerebellar vermis participates in a form of spinal facilitation that becomes apparent only after sexual experience has been acquired, though the effect in intact animals remains a trend requiring confirmation.

This interpretation is consistent with the established literature on cerebellar control of spinal reflexes. H-reflex operant conditioning depends on cerebellar integrity, and ablation of the cerebellar nuclei abolishes both the acquisition and the retention of this learning [13,22,23]. More directly, stimulation over the cerebellar vermis facilitates the soleus H-reflex, confirming that vermal output can raise the excitability of the spinal motoneuron pool [4]. The H-reflex is a sensitive readout of this state because its amplitude reflects not only motoneuron excitability but also the balance of Ia afferent transmission and the presynaptic and interneuronal control that operates under descending modulation [12]. The loss of H-wave facilitation after vermal lesions in our experienced animals is therefore consistent with the removal of a descending facilitatory influence that sexual learning had recruited. That the larger reduction followed the lobule 7 lesion and the smaller one the lobule 6a lesion argues against a uniform, non-specific consequence of tissue damage and points instead toward graded, region-related contributions. Such a graded pattern is consistent with the modular organization of the vermis into functionally distinct microzones, whose Purkinje cells are thought to bidirectionally modulate their output during learning in response to the directional and temporal demands of their downstream circuitry [7].

The M-wave produced an unexpected pattern. Sexually experienced animals showed shorter M-wave latencies than inexperienced ones (*p* = 0.026, d = 1.405), and M-wave amplitude was lower in lesioned experienced animals (Intact vs. L6a: *p* = 0.007, d = 1.957; Intact vs. L7: *p* = 0.003, d = 2.285), while remaining statistically unchanged in inexperienced animals. These observations require careful interpretation. The M-wave reflects the direct activation of motor axons and does not traverse the reflex arc or any descending pathway at the moment it is evoked, so an acute cerebellar lesion cannot plausibly reshape it through a direct neural route within a single recording session. The more parsimonious readings are that these differences reflect experience-associated changes in peripheral motor properties, possibly linked to the hormonal and trophic state that accompanies sexual activity [24,25], or that they arise from pre-existing variation between groups or from technical factors in stimulation and recording. We therefore present them as observations worth pursuing rather than as evidence of cerebellar control over the peripheral motor response, and they will require confirmation in a design that separates peripheral from central contributions.

The selective involvement of these lobules is characterized by their afferent organization. Lobules 6a and 7 of the posterior vermis are anatomically suited to integrate the sensory and motor signals generated during copulation. Both receive convergent multimodal input through two principal afferent systems: mossy fibers from the spinocerebellar tracts, which relay proprioceptive information from the hindlimbs and pelvis, together with pontocerebellar fibers carrying copies of corticomotor commands [6], and climbing fibers from the dorsal accessory olive, which deliver somatosensory error signals through the spino-olivary pathway [5,26]. Lobule 6a is reported to process somatosensory information at latencies of 9 to 12 ms, while lobule 7 contributes to sensorimotor integration over a low-frequency activation range [14,15,26,27]. The convergence of pelvic and perineal afferents onto lobule 6a, as shown directly in this model by Ortiz-Pulido and colleagues [16], provides the region with a plausible anatomical substrate for encoding the sensory consequences of copulatory movements, consistent with the activation of vermal granule cells reported during sexual behavior [14,15].

At the synaptic level, the most likely substrate for this learning is long-term depression at parallel fiber–Purkinje cell synapses, driven by the coincident activity of climbing and parallel fibers, as originally formulated in computational models of cerebellar plasticity [3], and demonstrated experimentally by Ito and colleagues [28]. Depression of Purkinje cell output disinhibits the cerebellar nuclei, increasing their facilitatory drive onto the brainstem and spinal circuits that set reflex gain [6,29]. The increase in H-wave amplitude we observed in experienced animals is compatible with a potentiation-like enhancement of Ia afferent–motoneuron transmission, the same direction of change targeted in operant up-conditioning of the H-reflex [22,30]. Cerebellar procedural memory is no longer thought to rest on a single mechanism. Alongside parallel fiber depression, learning-related changes in molecular-layer interneuron activity contribute to the stored output, and different cerebellar regions appear to recruit distinct types of plasticity [8,31], pointing to a more distributed cellular substrate than the classical model assumed.

These mechanistic interpretations must be considered alongside an important caveat. Copulation is not a pure motor act. It carries motivational, hormonal, sensory, autonomic, and reward-related components, any of which could alter spinal excitability through routes that bypass cerebellar motor learning altogether. Steroid hormones tied to sexual activity reorganize spinal motoneurons morphometrically and shift their firing thresholds [24,25], so part of what we attribute to cerebellar plasticity may run in parallel with these endocrine effects. Isolating the motor-learning component will require control conditions that the present design did not include, such as non-copulatory motor training, repeated exposure to a female without copulation, or yoked handling that reproduces the paradigm’s sensory and temporal structure without the full copulatory sequence.

Neither sexual experience nor the lesions altered H-wave latency or duration. The timing of the monosynaptic response was preserved, while its magnitude varied, suggesting that the cerebellum acts on the reflex gain rather than on the conduction or temporal synchronization of its underlying transmission. This dissociation is consistent with current accounts in which the cerebellum shapes motor output through predictive, feedforward control that adjusts the strength of motor circuits without rewriting the basic temporal architecture of the reflex arc [3,32,33,34,35].

Several limitations constrain the scope of these conclusions. First, assignment to the sexually experienced group was performance-based rather than randomized: animals reached SE status by meeting an ejaculation criterion across three independent sessions rather than through controlled exposure, so pre-existing differences in motor excitability, neuroendocrine responsiveness, or motivational state cannot be fully separated from the effects of learning itself. The exclusion of non-copulators and poor copulators, while necessary to define a coherent learning criterion, also restricts generalizability: the findings are limited to males with efficient copulatory performance, and whether the cerebellar facilitation described here scales with performance level or is present at reduced magnitude in subthreshold performers cannot be determined from the present data.

With six animals per group and no a priori power calculation, we rely on the 95% confidence intervals around the principal contrasts (Table 1) rather than on *post hoc* power: the two lesion effects show intervals that exclude zero, indicating adequate precision, whereas the interval for the intact-animal trend includes zero and marks that effect as one to confirm in a larger sample. Recordings were obtained under urethane, the standard agent for acute spinal reflex work because it preserves monosynaptic transmission with little distortion [20]; since every group received the same protocol, any attenuation of excitability is uniform rather than differential, although absolute amplitudes would likely be larger in conscious preparations [36,37]. The absence of a sham-lesion group means we cannot fully separate cortical ablation from the surgical procedure itself, although the graded difference between L6a and L7 outcomes and the confinement of lesion effects to experienced animals argue against a purely non-specific cause. Histological verification confirmed that lesions were confined to the target lobule in all animals included in the final analysis, with no evidence of spread to adjacent structures; individual-level morphometric quantification was not performed, and this should be addressed in future work using chemical or optogenetic approaches with sham controls.

These limitations suggest specific directions for future work: tracking molecular markers of potentiation at Ia afferent–motoneuron contacts and of depression at parallel fiber–Purkinje cell synapses in lobules 6a and 7, and clarifying the contribution of steroid hormones to the spinal changes, given the documented reorganization of motoneurons during sexual learning [24,25]. From a clinical standpoint, the link is suggestive but should not be overstated: operant conditioning of the H-reflex improves walking in people with incomplete spinal cord injury, with effects lasting for months [30,38,39,40], and cerebellar stimulation increases spinal motoneuron excitability in intact subjects [4]. Our data, obtained in anesthetized animals, cannot be extrapolated directly to rehabilitation; at most, they reinforce, as a distant conceptual implication, the idea that natural behavioral learning engages the kind of distributed cerebello-spinal plasticity that therapeutic conditioning seeks to harness, a possibility that awake preparations would be needed to test.

## 5. Conclusions

Sexual experience was associated with a near-significant trend toward higher soleus H-wave amplitude in intact animals; in the restricted analysis of experienced animals, electrolytic lesions of lobules 6a and 7 of the cerebellar vermis produced significant, graded reductions in that amplitude (larger for lobule 7) without equivalent effects in inexperienced controls. These findings indicate that the posterior vermis contributes to spinal facilitation that becomes apparent only after copulatory learning has been acquired.

The preservation of H-wave latency and duration across all conditions dissociates this modulation from axonal conduction properties and points to a cerebellar action on synaptic gain, consistent with the feedforward, predictive role attributed to vermal circuits in motor learning. Shorter M-wave latency in experienced animals suggests that sexual learning entails peripheral motor reorganization that accompanies these central changes. The present findings provide preliminary evidence that natural motor learning engages a form of cerebello-spinal plasticity involving lobules 6a and 7. This relationship will need to be confirmed and mechanistically characterized by adequately powered studies using molecular markers and behavioral controls. Because allocation to the experienced group was performance-based rather than randomized, such studies will also need to control exposure independently of outcome to isolate the contribution of sexual learning from pre-existing individual variation in motor and neuroendocrine phenotype.

## Figures and Tables

**Table 1:** *Post hoc* comparisons of H-wave amplitude between experimental groups. Planned contrasts testing specific hypotheses about the effects of sexual experience in intact animals and the impact of cerebellar lesions on learned facilitation.

Comparison	Estimate	SE	95% CI	df	t-Ratio	*p*-Value	Cohen’s d
Intact animals							
Inexperienced vs. Experienced	−0.700	0.328	[−1.44, 0.04]	9.11	−2.133	0.061	0.592
Experienced animals							
Intact vs. L6a Lesion	1.228	0.421	[0.125, 2.331]	14.0	2.913	0.029	1.450
Intact vs. L7 Lesion	1.910	0.453	[0.737, 3.082]	15.4	4.218	0.002	2.256
L6a vs. L7 Lesion	0.682	0.446	[−0.481, 1.845]	14.6	1.528	0.307	0.805

## Data Availability

The datasets generated and analyzed during the current study are available from the corresponding author on reasonable request.

## Abbreviations

The following abbreviations are used in this manuscript:

SI	Sexually inexperienced
SE	Sexually experienced
SI-I	Sexually inexperienced intact rats
SE-I	Sexually experienced intact rats
SI-L6a	Sexually inexperienced rats with lobule 6a lesion
SE-L6a	Sexually experienced rats with lobule 6a lesion
SI-L7	Sexually inexperienced rats with lobule 7 lesion
SE-L7	Sexually experienced rats with lobule 7 lesion
NC	Non-copulator
PC	Poor copulator
L6a	Lobule 6a of the cerebellar vermis
L7	Lobule 7 of the cerebellar vermis
EMG	Electromyographic
H-reflex	Hoffmann reflex
REML	Restricted Maximum Likelihood
Hmax/Mmax	Ratio of maximum H-reflex amplitude to maximum M-wave amplitude
